# Efforts to alter the trajectory of neonatal mortality in Malawi: evaluating relative effects of access to maternal care services and birth history risk factors

**DOI:** 10.7189/jogh.08.020419

**Published:** 2018-12

**Authors:** Bareng AS Nonyane, Emmanuel Chimbalanga

**Affiliations:** 1 Department of International Health, Johns Hopkins Bloomberg School of Public Health, Baltimore, Maryland, USA; 2 USAID’s ONSE Health Activity, Management Sciences for Health (MSH), Lilongwe, Malawi

## Abstract

**Background:**

The neonatal mortality rate (NMR) in Malawi has remained stagnant at around 27 per 1000 live births over the last 15 years, despite an increase in the uptake of targeted health care interventions. We used the nationally representative 2015/16 Demographic Health Survey data set to evaluate the effect of two types of maternal exposures, namely, lack of access to maternal or intra-partum care services and birth history factors, on the risk of neonatal mortality.

**Methods:**

A causal inference approach was used to estimate a population attributable risk parameter for each exposure, adjusting for co-exposures and household, maternal and child-specific covariates. The maternal exposures evaluated were unmet family planning needs, less than 4+ antenatal care visits, lack of institutional delivery or skilled birth attendance, having prior neonatal mortality, short (8-24 months) birth interval preceding the index birth, first pregnancy, and two or more pregnancy outcomes within the preceding five years of the survey interview.

**Results:**

We included 9553 women and their most recent live birth within 3 years of the survey. The sample’s overall neonatal mortality rate was 18.5 per 1000 live births. The adjusted population attributable risk for first pregnancies was 3.9/1000 (*P* < 0.001), while non-institutional deliveries and the shortest preceding birth interval (8-24 months) each had an attributable risk of 1.3/1000 (*P*s = 0.01). Having 2 or more pregnancy outcomes within the last 5 years had an attributable risk of 3/1000 (*P* = 0.006). Attending less than 4 ANC visits had, a relatively large attributable risk (2.1/1,000), and it was not statistically significant at alpha level 0.05.

**Conclusions:**

Our analysis addresses the gap in the literature on evaluating the effect of these exposures on neonatal mortality in Malawi. It also helps inform programs and current efforts such as the Every Newborn Action 2020 Plan. Increasing access to maternal care interventions has an important role to play in changing the trajectory of neonatal mortality, and women who are at an increased risk may not be receiving adequate care. Recent studies indicate an urgent need to assess gaps in service readiness and quality of care at the antenatal and obstetric care facilities.

The neonatal mortality rate (NMR) in Malawi has remained stagnant at around 27 per 1000 live births over the last 15 years [1-6]. This is despite the decrease in overall under-5 mortality rates and an increase in the uptake of targeted health care interventions such as antenatal care (ANC) visits, increased availability of obstetric and delivery services or increased uptake in facility delivery or skilled birth attendants, postnatal visits for mother and baby [3,7]. A number of challenges still remain in Malawi. Recent studies indicate that birth asphyxia, prematurity and infections account for most of the neonatal deaths [8] and these can be reduced with proper pre-, intra- and post-partum care. There is still low coverage of some of the interventions, for example, only 51% of mothers attended 4 or more ANC visits according to the 2015/16 Demographic and Health Survey (DHS) [3] and 44% according to the Multiple Indicator Cluster Survey (MICS) [9]. Furthermore, even with increased access to health care, service quality and facility readiness are still inadequate [10-19].

Studies using summary data at national levels have shown that improved program efforts can lead to a reduction in preventable neonatal mortality rates in low-income countries in order to make them comparable to those in more industrialized countries. Bhutta et al 2014 [7] used the Lives Saved Tool (LiST) to conduct an analysis of the potential impacts and costs of such efforts using country-level data. One of the main findings from this analysis was that worldwide, 71% (1.9 million) of neonatal deaths per year could be averted through 100% coverage and improved quality of interventions before and during pregnancy, as well as during the intra-partum and post-partum periods. A systematic review of studies from low and middle income countries reported that in 10 out of 19 studies evaluated, there was a significant risk reduction in neonatal mortality among those who delivered at a facility, with a relative risk meta-estimate of 0.71 (95% CI = 0.54, 0.87). [20]. Another systematic review in 2014, however, found no evidence that facility delivery had a significant effect on perinatal mortality (odds ratio (OR) = 1.21, 95% CI = 0.79-1.84) [21].

In addition to health care interventions or service related factors, there are maternal and child biological and socio-demographic factors, as well as household risk factors that have been well documented. For example, male sex is associated with an increased risk, and neonatal mortality tends to cluster within a small subset of mothers and those who have high fertility [22-24]. Examples of socio-demographic factors include the mother’s educational status which may affect timely care-seeking for antenatal care or for neonatal infections [11, 25-27]. Another contributor to NM risk is related to unmet need for family planning services which can lead to short inter-pregnancy intervals that confer a high risk of NM [28]. This has also been highlighted by the Lancet Every Newborn Study Group as essential component of improving newborn survival [29, 30]. In Malawi, demand for family planning needs is met for only 76% of married women and 53% of sexually-active unmarried women aged 15 to 49 years [3].

Most evaluations of the effects of health care interventions for reducing NMR in Malawi have been done on a small scale within a few districts, or focusing on only one or two interventions [1,10,11,31]. An analysis using the Malawi 2004 DHS showed that those who lived closer to the facility were more likely to deliver there than at home, but facility delivery was not associated with a reduction in the risk of early neonatal mortality [32]. Malawi’s national data on summary measures of coverage of neonatal and maternal care interventions have been included in international evaluations of potential impact for these and averaging across countries [2,7]. There is a gap in literature that uses a nationally representative data to jointly evaluate the effects of different types of exposures on the risk for NM in Malawi.

The aim of this study was to add to the literature using more recent data from the Malawi 2015/16 DHS. We applied a causal inference approach to individual mother-level data to evaluate the relative effects of two types of risk exposures, namely, lack of access to maternal care and intra-partum services and prior birth history events, on the risk of neonatal mortality. The causal inference approach used here enables us to adjust the attributable risk estimate of each exposure for the effect of its co-exposures, as well as household, maternal and child-specific covariates. The results of this analysis can help guide current efforts to reduce neonatal mortality such as the Every Newborn Action Plan [18] and to help achieve sustainable development goals [33,34].

## METHODS

### Data and variables definition

**Figure 1:**
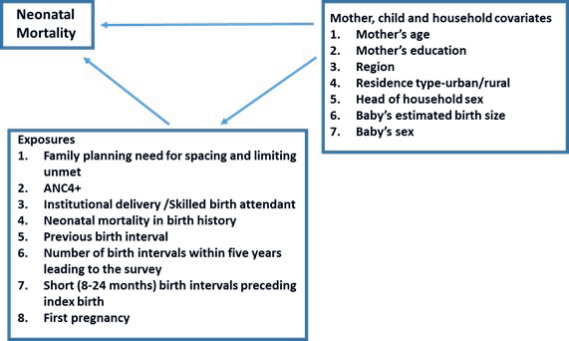
Conceptual diagram for the population intervention models.

Data about each participating woman’s most recent pregnancy and live birth (within 3 years leading to the survey) were extracted from the Malawi DHS 2015/16. Thus, all information was based on the woman’s ability to recall the health care services accessed and their birth history. Various combinations of the following risk exposures were evaluated (**Figure 1**): having unmet family planning needs for spacing and limiting; not having four or more antenatal care visits (ANC4+), lack of institutional delivery (Ideliv) or skilled birth attendance, having experienced prior neonatal mortality, short (8-24 months vs longer or first pregnancy) birth interval preceding the index birth, first pregnancy vs second or more, two or more pregnancy outcomes within the five years of the survey interview.

Maternal, child and household covariates included the newborn’s sex, preceding pregnancy interval, mother’s age and education, household socio-economic status, residence type (urban/rural) and region (North, Central and South). The DHS followed a two-stage stratified sampling design where each of the 28 districts in Malawi was stratified into urban or rural, and within those strata, the standard enumeration areas were sampled proportional to size.

### Analytical approach

In this analysis, we fitted population intervention models (PIM) which employ a causal inference approach to determine the relative importance of lack of access to different maternal and newborn-care interventions on the risk of neonatal mortality. Theoretical underpinnings of this approach have been extensively described by Hubbard, van der Laan and Gruber [35-37] and an R implementation of this is in the package *multiPIM* by Ritter et al (2014) [38]. In order to briefly describe the approach here, we first define the components of our data as follows. Y is the outcome which is a binary indicator of neonatal death for the most recent live birth*;* A denotes the exposure so that A = 0 if the woman is unexposed (that is, accessed care or has a low risk birth history), and A = 1 if exposed; **W** is a set of household, mother and child covariates of various types. Since there are multiple exposures in our current study, A is an element of a matrix **A** where rows correspond to the individual women and columns correspond to the exposures. Likewise, **W** is a matrix of covariates. The causal inference approach assumes that intervention effects have a common model G: *g(Ai = 0| Wi)* which gives the predicted probabilities of being in the low risk category *Ai = 0* given a vector of covariates Wi. G is widely known as the *propensity score* and oftentimes, it is simply modeled by a logistic regression for the binary intervention or exposure of interest. A model for the outcome Y is denoted by *Q(A = a,W),* and this can take on various functional forms depending on the distribution of Y.

Under the causal inference assumptions [38], the PIM approach estimates a target population averaged causal parameter φ which is the difference between the overall mean of *Y* and the mean of the outcome among participants who are unexposed [A = 0], averaged over the covariates:

φ = *Ê* [Y_0_] – *Ê* [Y]

In other words, for a *j*^th^ a exposure φ^j*^  is the amount of the outcome that would have been averted if everyone was unexposed to exposure *A*^j*^. In that sense, the PIM parameter is the reverse of the population attributable risk which is traditionally used in in epidemiology studies.

In the derivation of the potential effects of each exposure, we adjusted for other co-exposures in addition to household, mother and child covariates as well as the DHS sampling weights in the intervention model g(0|W). We present the φ parameter alongside its estimated standard error and the *P* associated with the test of the null hypothesis that its true value is 0. One of the main advantages of following this approach that is worth noting here is the flexibility to specify different candidate parametric and non-parametric models for estimating g(0|W) and Q(0,W). The best among these is selected via *v*-fold cross-validation. This is referred to as the ‘super learner’ approach. We estimated our TMLE parameters with logistic regression for the exposure (g) models and a nonparametric recursive partitioning for the outcome (Q) models.

Analysis was carried out with 3 combinations of the risk exposures due to a strong correlation (hence complete confounding) between birth intervals and the number of pregnancy outcomes in the past 5 years, and between institutional delivery and skilled birth attendance; and also because of small sample sizes in the high risk categories of previous neonatal mortality and shortest birth intervals. With small sample sizes, different stratifications led to some categories having probabilities that were completely determined (all 0s or 1s).

## RESULTS

**Table 1** lists the characteristics of 9553 women who participated in the DHS survey and had a live birth within 3 years leading to the survey. **Table 2** shows the different exposures and the corresponding unadjusted estimates of the risk of neonatal death. The overall neonatal mortality rate was 18.5 per 1000 live births, and this varied by categories (high or low risk) of each of the exposures.

**Table 1:** Characteristics of the participants – women and newborns from the most recent live birth within 3 years of the survey

	N	%
**Total**	**9553**	**100**
**Region:**		
North	1667	18.17
Central	3182	34.69
South	4323	47.13
**Residence type:**		
Urban	1462	15.94
Rural	7710	84.06
**Mother’s age group (years):**		
15-19	1009	11
20-24	2892	31.53
25-29	2056	22.42
30-34	1649	17.98
35-39	1051	11.46
40-49	515	5.61
**Mother's education:**		
No education	1037	11.31
Primary	6129	66.82
Secondary	1841	20.07
Higher	165	1.8
**Head of household sex:**		
Male	6945	75.72
Female	2227	24.28
**Household socio-economic status:**		
Poorest	2107	22.97
Poorer	2007	21.88
Middle	1802	19.65
Richer	1672	18.23
Richest	1584	17.27
**Facility delivery:**		
No	596	6.5
Yes	8576	93.5
Child sex:		
Male	4626	50.44
Female	4546	49.56
**Child's weight estimate based on recall:**		
Very large	783	8.54
Than average	2334	25.45
Average	4610	50.26
Than average	1073	11.7
Very small	372	4.06
**Preceding birth interval (months):**		
8-24	715	7.8
25-36	1724	18.8
37-50	1906	20.78
51-226	2460	26.82
First pregnancy	2367	25.81

**Table 2:** Exposures and the associated neonatal mortality rates by category

	N	NMR/1000
**Total**	**9553**	**18.5**
**Facility delivery**		
No	608	41.2
Yes	8945	18.3
**ANC 4+:**		
No	4742	22.7
Yes	4811	17.1
**Skilled birth attendant:**		
No	751	40.3
Yes	8802	17.9
**Two + pregnancy outcome in the last 5 years:**		
No	6074	16.6
Yes	3479	25.6
**Shortest birth interval:**		
>24 months	8795	18.4
8-24 months	758	37.2
First pregnancy:		
No	7078	17.8
Yes	2475	25.6
**Previous NNM:**		
No	9472	19.9
Yes	81	24.1

NMR – neonatal mortality rate, ANC – antenatal care, NNM – neonatal mortality

The exposures that had the least proportions of exposed mothers during their most recent pregnancy were ANC4+ visits (50%), two or more births under 5 in the last 5 years (36%), first pregnancy(26%). Institutional delivery / SBA and the shortest birth interval of 18-24 months had the least number of exposed women (**Table 3**). The naive population attributable risk (that is, the difference between the proportion of those who were in the low risk category and had a neonatal death, and the overall NM rate) ranged from 0 to 3 per 1000 live births that could have been protected from the risk of neonatal death if everyone had been unexposed (**Table 3**).

**Table 3:** Unadjusted estimates of exposures and associated population attributable risk*

	Proportion in the exposed group	Proportion in the unexposed group with NNM	Proportion in exposed group with NNM	Naive population attributable risk proportion	Naive population attributable risk rate(per 1000)
Institutional delivery	0.064	0.0172	0.0378	-0.0013	-1.3
ANC4+	0.496	0.0168	0.0202	-0.0017	-1.7
SBA	0.079	0.0169	0.0373	-0.0016	-1.6
Family planning need unmet	0.166	0.0173	0.0245	-0.0012	-1.2
Two or more births under-5	0.364	0.0158	0.0233	-0.0027	-2.7
Shortest (<24 months) birth interval	0.079	0.0175	0.0303	-0.001	-1.0
First pregnancy	0.259	0.0165	0.0242	-0.002	-2.0
Previous NNM	0.009	0.0184	0.0370	-0.0001	-0.1

NMR – neonatal mortality rate, ANC – antenatal care, NNM – neonatal mortality, SBA – skilled birth attendance

*Attributable risk estimates expressed as proportions as well as deaths per 1000 live births potentially averted. Proportions are rounded to 3 decimal places.

**Table 4** shows the adjusted TMLE estimates of the causal parameters φ^j*^ . The adjusted estimates of the population attributable risk parameter ranged from 0 to 3.9 per 1000 live births that would have potentially been protected from neonatal deaths had everyone been in the low risk category. In the first combination of exposures (Exposure Set A), first pregnancies were associated with the highest-risk of 3.9 per 1000 live births (*P* < 0.001). Having less than 4 ANC visits had the second largest attributable risk of 2.1 per 1000 live births because of the high prevalence of this exposure but this was not statistically significant after adjusting for covariates and co-exposures. Non-institutional delivery and the shortest birth interval (8-24 months) each had a population attributable risk of 1.3 per 1000 live births (*P*s = 0.01). In a sensitivity analysis where SBA was used instead of institutional delivery, the population attributable risk for non-SBA was 1.6 per 1000 live births (*P* = 0.005). In a sensitivity analysis where first pregnancy was added without the shortest birth interval, the population attributable risk for first pregnancy was 2.9 per 1000 live births (*P* = 0.02).

**Table 4:** Attributable risk parameter estimates: expressed as deaths per 1000 live births that could have potentially been averted had everybody been in the unexposed category

Exposure Set A				
**Exposure**	**Parameter estimate**	**Standard error**	**Test statistic**	***P-*value**
Institutional delivery	-1.3	0.5	2.550	0.011
ANC4+	-2.1	1.4	1.514	0.130
Family planning need unmet	-1.2	0.7	1.760	0.078
Shortest (<24 months) birth interval	-1.3	0.5	2.601	0.009
First pregnancy	-3.9	1.1	3.411	0.001
**Exposure Set B**				
**Exposure**	**Parameter estimate**	**Standard error**	**Test statistic**	***P-*value**
Institutional delivery	-1.3	0.5	2.506	0.012
ANC4+	-1.9	1.4	1.376	0.169
Family planning need unmet	-1.2	0.7	1.721	0.085
Two or more births under-5	-3.2	1.1	2.855	0.004
**Exposure Set C**				
**Exposure**	**Parameter estimate**	**Standard error**	**Test statistic**	***P-*value**
Institutional delivery	-1.3	0.5	2.595	0.001
ANC4+	-2.1	1.4	1.495	0.135
Family planning need unmet	-1.2	0.7	1.787	0.074
Previous NNM	-0.2	0.2	0.958	0.338

NNM – neonatal mortality, ANC4+ – four or more antenatal care visits

In Exposure Set B, first pregnancy and birth interval were removed and replaced by having 2 or more pregnancy outcomes within the last 5 years of the survey and this had a population attributable risk of 3 per 1000 live births (*P* = 0.004). In Exposure Set C, having previous neonatal mortality had a negligible population attributable risk 0.2 per 1000 live births (*P* = 0.34). In all three models, having unmet family planning needs for spacing or limiting was found to have an attributable risk of 1.2 /1000 (*P*s = 0.08). In sensitivity analyses where birth history was excluded, the population attributable risk of having family planning needs unmet remained the same.

## DISCUSSION

We have conducted an evaluation of the effect of not accessing some maternal and intra-partum care services, and individual’s prior birth history on the risk of NM in Malawi. Lack of institutional delivery or skilled birth attendance, and pregnancy history birth history evaluations at ANC visits have significant roles to play. To our knowledge, these two types of exposures have not been evaluated within the same analysis for their relative effects on the risk of NM in Malawi. Our analysis addresses this existing gap in the literature, and it provides valuable information to help identify interventions that have a potential to change the trajectory of NM if they were scaled up. We note that the significant effect of birth history factors on the risk of NM likely indicates that women who are at an increased risk may not be receiving adequate care. This may be due to the gaps that still exist in the service readiness and quality of implementation of the existing WHO recommendations for family-planning and antenatal care services [12-19].

Our findings with respect to birth history factors are similar to what has been reported in the literature, which shows an elevated risk among first time mothers, and for births preceded by short inter-pregnancy / inter-birth intervals [39-46]. Furthermore, the finding of an elevated risk for high fertility mothers is similar to that of Kozuki et al (2013) [28] who found that among mothers with high fertility, there was an elevated risk of NM for all children irrespective of birth order, after adjusting for socioeconomic and reproductive health factors. This, they noted, suggest some residual confounders that explain a specific type of risk suffered by children born to high fertility mothers. One could also argue that this may be reverse causality in a sense that mothers who experience neonatal deaths may be more likely to try and have more children. Our findings with respect to institutional delivery contradict a finding from a study using the Malawi 2004 DHS data [32] in which facility delivery was not shown to be associated with a reduced risk on NM.

The population attributable risks associated with lack of access to institutional delivery/skilled birth attendance and birth history factors, though statistically significant, are relatively small compared to the overall NMR, and this is further evidence that there are some residual effects of unmeasured covariates such as maternal biological factors or household factors as well as quality of service at facilities which lead to clustering on NM within a small subset of mothers [23]. Furthermore, care-seeking for early neonatal illness is often delayed or not practiced in some low-income settings due to various social and economic factors [6,11,25,47,48]. Hence there is still a need for programs to identify and target high risk mothers who have had adverse events in the past or who may be less likely to seek care for neonatal illness. We also note that the ability to identify and address the needs of high risk mothers is highly dependent on adequate service quality and facility readiness. The most recent literature indicates that this is still lacking in Malawi [12]. Our future work will involve an assessment of service readiness for ANC and obstetric care from the perspective of the mother given at exit interviews.

The multi parameter intervention models approach used here offers an efficient way to apply machine learning algorithms to evaluate the marginal effects of the exposures, adjusting for co-exposures and for baseline covariates. The availability of the R implementation of this approach offers researchers a vehicle to apply this in exploratory analysis where there could potentially be large numbers of interventions and covariates. The causal parameter has an interpretation akin to the widely used population attributable risk, and it can be derived using the traditional semi-parametric and non-parametric models as the foundation for estimating the propensity score (g) models and the outcome (Q) models.

One of the limitations of our study is that our data on access to different interventions and birth history services is dependent on the participating mother’s ability to recall the events. We limited this by only evaluating data from the most recent births within 3 years of the survey. Another potential problem is that there may be misclassification of neonatal deaths and stillbirths [49]. The multiPIM package does not yet support methods that include missing values in some of the predictors. The interventions related to vaccinations and medications received during pregnancy were also considered but they had large numbers of missing values which made it difficult to conduct reliable estimates.

## CONCLUSIONS

Our analysis addresses the gap in the literature on evaluating the effect of these exposures in Malawi, and it helps inform programs and current efforts such as the Every Newborn Action 2020 Plan. It shows that increasing access to maternal care interventions has an important role to play in changing the trajectory of neonatal mortality. Birth history factors play an important role on the risk of NM and we hypothesize that women who are at an increased risk may not be receiving adequate care. Recent studies indicate that gaps still exist in service readiness and quality of implementations of the existing WHO-recommendations for family planning and antenatal care services, as indicated by recent studies.
